# Antibacterial, Antibiofilm, and Antiviral Farnesol-Containing Nanoparticles Prevent *Staphylococcus aureus* from Drug Resistance Development

**DOI:** 10.3390/ijms23147527

**Published:** 2022-07-07

**Authors:** Aleksandra Ivanova, Kristina Ivanova, Luisa Fiandra, Paride Mantecca, Tiziano Catelani, Michal Natan, Ehud Banin, Gila Jacobi, Tzanko Tzanov

**Affiliations:** 1 Group of Molecular and Industrial Biotechnology, Chemical Engineering, Universitat Politécnica de Catalunya, 08222 Terrassa, Spain; aleksandra.asenova@upc.edu (A.I.); kristina.ivanova@upc.edu (K.I.); 2 Department of Earth and Environmental Sciences, Research Center POLARIA, Universita degli Studi di Milano-Bicocca, 20900 Milano, Italy; luisa.fiandra@unimib.it (L.F.); paride.mantecca@unimib.it (P.M.); 3 Interdepartmental Microscopy Platform, University of Milano-Bicocca, 20126 Milano, Italy; tiziano.catelani@unimib.it; 4 The Institute for Advanced Materials and Nanotechnology, The Mina and Everard Goodman Faculty of Life Sciences, Bar-Ilan University, Ramat-Gan 52900, Israel; natan.michal@gmail.com (M.N.); ehud.banin@biu.ac.il (E.B.); gilajacobi@gmail.com (G.J.)

**Keywords:** farnesol nanoparticles, bacterial eradication, biofilm prevention and elimination, biocompatibility, SARS-CoV-2

## Abstract

Multidrug antimicrobial resistance is a constantly growing health care issue associated with increased mortality and morbidity, and huge financial burden. Bacteria frequently form biofilm communities responsible for numerous persistent infections resistant to conventional antibiotics. Herein, novel nanoparticles (NPs) loaded with the natural bactericide farnesol (FSL NPs) are generated using high-intensity ultrasound. The nanoformulation of farnesol improved its antibacterial properties and demonstrated complete eradication of *Staphylococcus aureus* within less than 3 h, without inducing resistance development, and was able to 100% inhibit the establishment of a drug-resistant *S. aureus* biofilm. These antibiotic-free nano-antimicrobials also reduced the mature biofilm at a very low concentration of the active agent. In addition to the outstanding antibacterial properties, the engineered nano-entities demonstrated strong antiviral properties and inhibited the spike proteins of SARS-CoV-2 by up to 83%. The novel FSL NPs did not cause skin tissue irritation and did not induce the secretion of anti-inflammatory cytokines in a 3D skin tissue model. These results support the potential of these bio-based nano-actives to replace the existing antibiotics and they may be used for the development of topical pharmaceutic products for controlling microbial skin infections, without inducing resistance development.

## 1. Introduction

The spread of microbial infections is an emerged concern, further aggravated by the appearance of antimicrobial resistance (AMR) to antibiotics and disinfectants [1]. Drug-resistant microbes are estimated to bring about 10 million deaths annually and reach an economic burden of $50 million by 2050 if no effective therapeutics are developed [1]. Additionally, bacteria often live in cell communities encased in an extracellular polymeric matrix (EPM), called a biofilm, creating additional antimicrobial tolerance [2]. Biofilm structures restrict drug penetration, increasing bacterial resistance by up to 1000-fold, compared to the planktonic cells [3,4]. *Staphylococcus aureus* (*S. aureus*) is an opportunistic Gram-positive pathogen that has been included in the WHO’s list of priority bacterial pathogens endangering human health [5] due to its ability to adapt to conventional antibiotics, rapidly developing multidrug resistance. This bacterium colonizes tissues and medical devices mostly in the form of biofilms, causing issues ranging from mild skin (e.g., boils, cellulitis, folliculitis) to life-threatening antibiotic-resistant infections (e.g., bacteremia, pneumonia, osteomyelitis, and device-related infections) [6,7]. The spread of this pathogen is of great concern and it is responsible for increased mortality, morbidity and a huge financial burden on the healthcare systems [8]. The potential of *S. aureus* to acquire resistance mechanisms quickly after the introduction of new antibiotics to the market, has called for the urgent need of more effective non-antibiotic alternatives for managing *staphylococcal* infections.

In this context, natural compounds found in essential oils, such as the terpenoid farnesol, have attracted attention as alternative actives to control microbial pathogens [9]. Farnesol, a sesquiterpene alcohol in essential oils, is a hydrophobic compound extracted from lemongrass, tuberose, balsam, citronella, cyclamen, and rose [10] with a demonstrated ability to inhibit the growth of medically relevant pathogens, and it is generally recognized as safe by the U.S. Food and Drug Administration [11]. Farnesol affects the cell membranes of bacterial species causing ionic imbalance and ion leakage, and consequently, cellular death [12]. This compound also interferes with the glycan rate of synthesis in the biofilm matrix, impairing the biofilm establishment [13]. Despite the promising therapeutic efficacy of farnesol against bacterial cells and their biofilms, its hydrophobicity and poor retention have restricted its medical application [14]. 

Recent advances in the field of nanotechnology allows the drawbacks of low drug solubility and undesirable interactions to be overcome, and can improve the drug therapeutic index [15]. Nano-sized materials and novel drug delivery systems have demonstrated that they can decrease the side effects of the actives, to possess stronger antibacterial properties and multivalent interaction with biological systems and cells, improving the bioavailability of therapeutic compounds, compared to the bulk solutions, due to the unique chemical and physical properties provided by the nanoform [16,17,18]. In contrast to the conventional antibiotics, the nanoformulated actives are able to penetrate the EPM of biofilms and eradicate the biofilm-encased bacterial cells. Previously, we found that the nanoformulation of antimicrobials improved their interaction with the bacterial membranes, and subsequently enhanced their antibacterial efficiency at a lower dosage than their bulk counterparts [16,17,19,20]. Furthermore, nano-sized antibiotics have demonstrated their ability to effectively penetrate the EPM and reduce mature biofilms [17]. 

**Figure 1:**
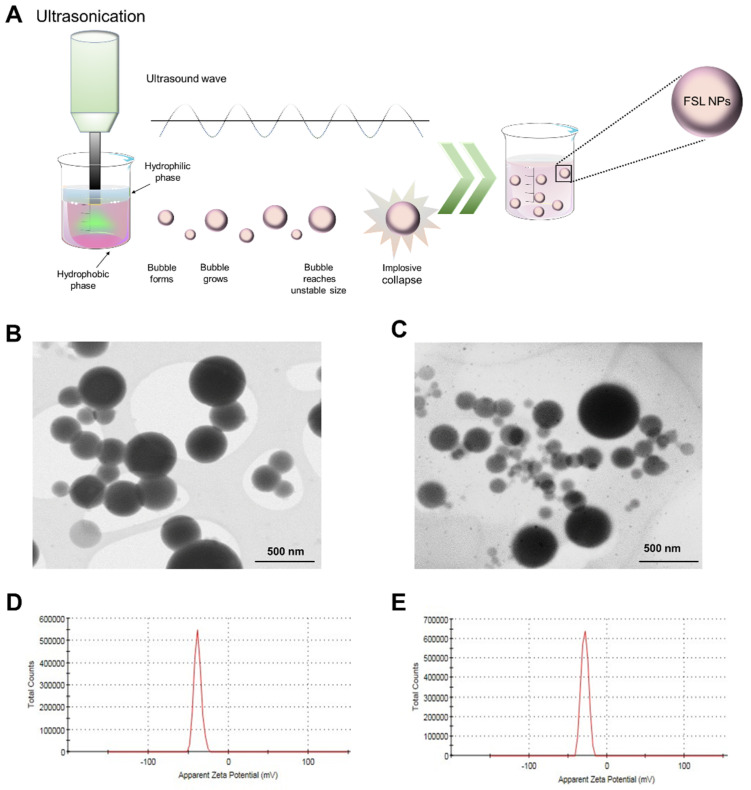
NP preparation and characterization. (**A**) Schematic representation of FSL NP synthesis using US. STEM images with magnification 120,000× of (**B**) empty NPs and (**C**) FSL NPs. Zeta−potential distribution graph showing negative zeta-potential values for (**D**) empty and (**E**) FSL NPs.

Herein, we aimed to engineer novel natural-based nanoformulations with enhanced antimicrobial activity against *S. aureu*s using a biocompatible monobutyl ester of poly (methyl vinyl ether/maleic) acid as a carrier in a one-step high-intensity ultrasound (US) process (Figure 1A). The use of nanoscale matter will provide enhanced interaction with the *S. aureus* cell membrane and result in easier penetration into the biofilms, increasing the antibacterial efficacy of nanoformulated farnesol at lower dosages, while lowering the risk of development of AMR. Moreover, the ongoing, severe, acute respiratory syndrome, COVID-19 (SARS-CoV-2), pandemic has outlined the global AMR crisis and the importance of developing highly-effective antibiotic-free antimicrobial strategies for the control of microbial contamination with a lower risk of resistance development [21]. Owing to the lipophilic nature of farnesol, the developed nanomodalities are also expected to intercalate into the lipid double layer of viral pathogens such as SARS-CoV-2 and inhibit the interaction of spike proteins with angiotensin-converting enzyme 2 (ACE2) proteins present on the host cells. The antimicrobial and antibiofilm activities of the developed nanoentities will be evaluated and compared to the bulk farnesol solution. The potential of the developed nano-entities to avoid the risk of resistance development will be further validated. Finally, the inflammation induction and cytotoxicity in a 3D skin tissue model will be assessed. 

## 2. Results and Discussion

### 2.1. NP Characterization

High intensity US was employed for the preparation of FSL NPs (Figure 1A) upgrading a patented self-assembly nanoencapsulation technology based on poly (methyl vinyl ether/maleic acid) and nonvolatile solvent propylene glycol [22]. The determined zeta-potential values of the empty NPs (without farnesol) and FSL NPs were −38.2 ± 4.46 mV and −28.3 ± 4.53 mV, respectively (Figure 1D,E), meaning high colloidal stability without the addition of surfactants. Generally, negative and positive zeta-potential values > ±20 mV are desirable to reach steric and electrostatic stabilization of NP suspension [23]. The obtained NPs demonstrated long-term stability and did not precipitate for up to 6 months in storage at room temperature. The negative charge of the FSL NPs is due to the carboxyl groups [24] in the polymer used for NP formation. 

NTA further determined the concentration of the empty and FSL NPs to be 8.03 × 10^10^ NPs mL^−1^ and 1.86 × 10^10^ NPs mL^−1^, respectively. The obtained STEM images confirmed the uniform spherical shape and smooth surface of the empty and FSL NPs with an average size between 130 and 150 nm (Figure 1B,C). Although the images display very polydisperse NPs, STEM is not suitable for the polydispersity characterization since it gives a local picture, which is not always representative. Therefore, the polydispersity index (PDI) of FSL NPs and empty NPs was measured using dynamic light scattering, which offers good statistics with respect to the in situ measurements of the size and PDI of nanomaterials. The PDI of the developed empty and FSL NPs was determined to be 0.2. Such PDI values are common for the polymer-based NPs and are considered acceptable in practice [25]. The amount of farnesol loaded into the NPs (5 × 10^9^ NPs mL^−1^) was determined to be 200 µg mL^−1^ using HPLC (Figure S1). 

### 2.2. Antibacterial Efficiency of FSL NPs against Planktonic Bacteria

*S. aureus* is a persistent pathogen, involved in the most common high-mortality and morbidity nosocomial and foreign-body-related infections treated with conventional antibiotics [26]. However, *S. aureus* strains are notorious for their natural capability to develop resistance to every antibiotic that has been discovered [7] and the development of efficient therapies has become a significant challenge for the scientific community. 

**Figure 2:**
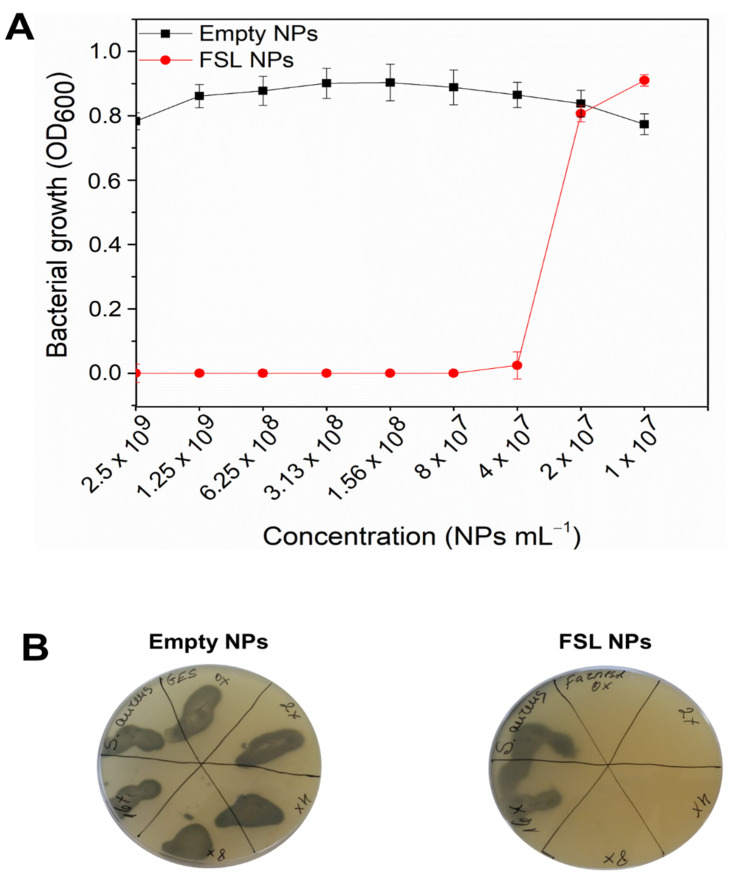
(**A**) *S. aureus* growth inhibition after 24 h incubation with empty and FSL NPs at different concentrations. (**B**) Bacterial survivor and representative images after culturing the samples on specific agar plates.

The novel FSL NPs demonstrated very strong bacterial growth inhibition (Figure 2A) at 4 × 10^7^ NPs mL^−1^ minimum inhibitory concentration (MIC) (0.8 µg mL^−1^), and 3.13 × 10^8^ NPs mL^−1^ (6.25 µg mL^−1^) minimum bactericidal concentration (MBC) (Figure 2B). After plating on selective agar and incubation for 24 h at 37 °C, the MBC was determined as the lowest concentration of the FSL NPs where no survived bacteria were observed. The empty NPs were not able to inhibit the bacterial growth, confirming that the bactericidal properties of the NPs are due to the presence of farnesol in the NPs. Moreover, the nano-formulated farnesol improved the bacterial growth inhibition properties (~100% growth inhibition), compared to its bulk solution (Figure S2) where higher concentrations were required. Although the antibacterial potential of sesquiterpene alcohol against *S. aureus* has been already described [12], the enhancement of its efficacy upon nanotransformation has not yet been reported. The hydrophobic nature of farnesol facilitates its accumulation in the bacterial membrane [12], causing cell leakage and death. In fact, it was reported that the exposure to terpene alcohols affects the cell membranes of several human pathogens, resulting in the leakage of K^+^ ions from cells [27], playing a key role in the maintenance of membrane potential, physiology, enzyme activation, and protein synthesis [28]. Moreover, the nanoform increased the interaction of the active with the bacterial cell wall membrane and improved its membrane penetration [17,20]. The Gram-positive bacterial membrane contains lipoteichoic acid with lipophilic ends that can “infiltrate” the natural hydrophobic compounds into the bacterial cell [29].

**Figure 3:**
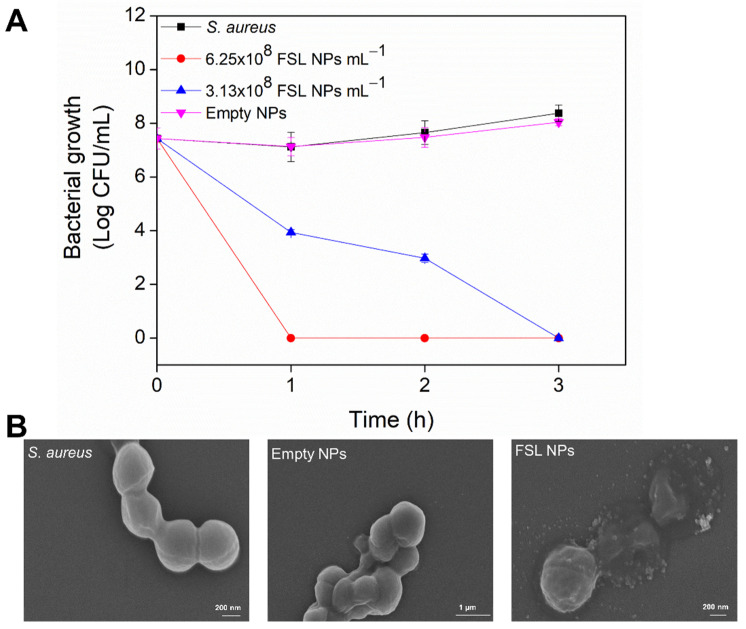
(**A**) Time−killing kinetics of empty and FSL NPs and the bacteria surviving after treatment for 1, 2, and 3 h. (**B**) SEM images of *S. aureus* without any treatment and incubated with empty or FSL NPs.

Time-killing kinetics of the FSL NPs at bactericidal concentrations were performed to assessed the viability of the treated bacteria (CFU mL^−1^) and evaluate the rate of bactericidal efficiency of the NPs. The bactericidal properties of the NPs towards *S. aureus* were found to be concentration- and time-dependent (Figure 3A). The FSL NPs at higher concentrations completely eliminated *S. aureus* (8 log reduction) within 1 h of contact. However, a longer incubation time (3 h) was required to eradicate the pathogen in the case of FSL NPs at the MBC, whereas the empty NPs did not affect the bacterial cell viability.

The interaction of the FSL NPs with the Gram-positive bacterium and the changes in the cell morphology and structure of the cells were further assessed using SEM (Figure 3B). *S. aureus* appear as smooth spheres with size ranging from 500 nm to 1 µm in diameter [30]. The exposure of the bacteria to empty NPs did not alter the bacterial cell morphology and the cell wall structure remained intact, similar to the control (non-treated bacteria). However, the treatment of *S. aureus* with FSL NPs at the MBC showed a pattern typical for a strong interaction between FSL NPs and the *S. aureus* membrane and strongly damaged the cells, which is associated with a disordered cytoplasmic structure, leakage of the cytoplasmic contents, and death. Such an unspecific mechanism of antibacterial action would exert less evolutionary pressure on the pathogen and avoid the occurrence of drug resistance [31]. The results from SEM are in agreement with the results obtained from the bacterial growth inhibition and time-killing kinetics assays.

Since the AMR is an emerging concern for health care, the potential of FSL NPs to trigger resistance development in Gram-positive *S. aureus* was tested relative to the antibiotic ciprofloxacin. The MIC value of ciprofloxacin increased sharply over the course of 30 days with a jump of 735. On the contrary, the MIC of FSL NPs increased slightly by only a factor of 5.25 when tested with *S. aureus*. Hence, FSL NPs present a huge advantage in terms of their use and the prevention of resistance among pathogenic bacteria.

### 2.3. Antibiofilm Activity

Since *S. aureus* bacteria often grow in biofilms on living tissues and medical devices, and these sessile communities are considerably less susceptible to antibiotics, much effort has been dedicated to developing novel antibiofilm therapeutics [32]. Farnesol nanoentities are expected to impair the biofilm establishment and reduce the already formed biofilm structures. The antibiofilm properties of FSL NPs were assessed using crystal violet to determine the total biofilm mass, SEM to visualize the biofilm formation, and LIVE/DEAD BacLight assays. 

#### 2.3.1. Inhibition of *S. aureus* Biofilm Formation

**Figure 4:**
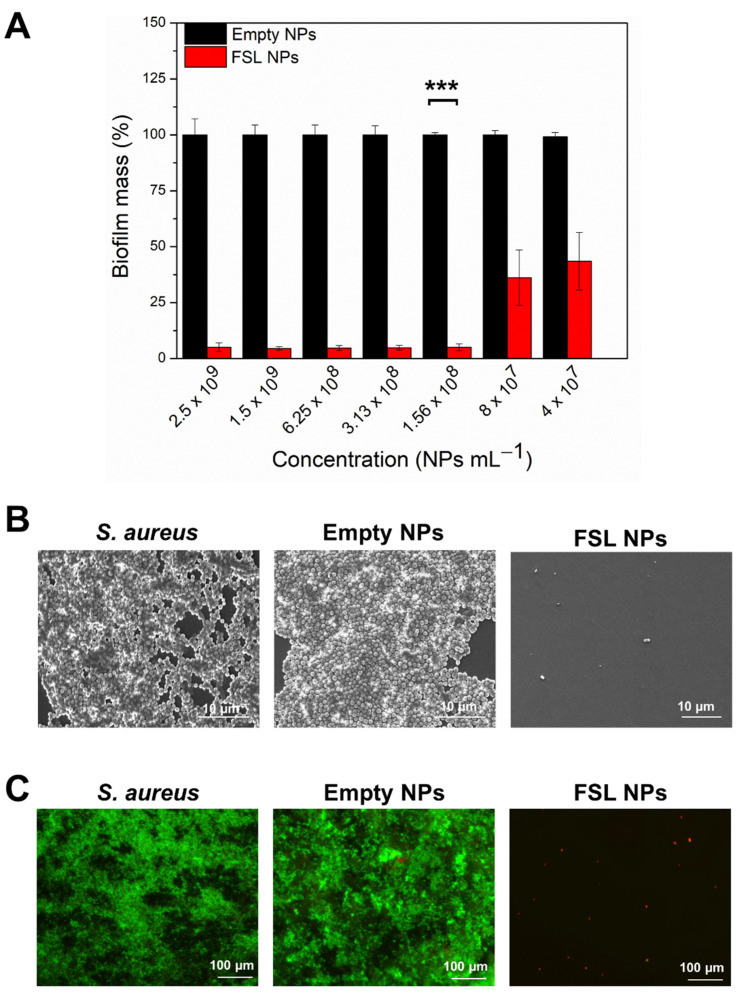
Inhibition of formation of *S. aureus* biofilm. (**A**) Total biofilm quantification determination of the NPs using crystal violet assay. (**B**) SEM observation of established biofilm with and without incubation with empty and FSL NPs at the same concentration (1.56 × 10^8^ NPs mL^−1^). (**C**) LIVE/DEAD BacLight kit microscopic visualization of treated and non-treated *S. aureus* biofilm with developed NPs at the same concentration (1.56 × 10^8^ NPs mL^−1^). The green and red fluorescence images are overlaid. The dead bacteria are stained in red and live bacteria are stained in green. Stars represent the statistical differences between the biofilm inhibition activities of FSL NPs at MBIC and empty NPs (*p* < 0.0001).

The FSL NPs demonstrated a strong capacity to impede the formation of the *S. aureus* biofilm (Figure 4A). Previous studies have shown that farnesol altered the rate of glucan synthesis, affecting the EPM accumulation by *S. aureus* [14] and compromising the cell membrane integrity [33]. Herein, the FSL NPs completely inhibited the *S. aureus* biofilm formation at very low concentrations of the active, compared to the bulk solution, due to the improved interaction between farnesol and the bacterial membrane and its enhanced antibacterial properties (Figure S3). The minimum biofilm inhibitory concentration (MBIC) of FSL NPs was determined to be 1.56 × 10^8^ NPs mL^−1^. As expected, the empty NPs did not impede the biofilm establishment, confirming that the antibiofilm effect was due to the presence of farnesol in the NPs.

The SEM images demonstrated that the *S. aureus* formed well-established biofilm structures, not affected by the empty NPs, whereas the FSL NPs reduced the bacterial cell viability and impaired biofilm formation (Figure 4B). These results were confirmed by fluorescence microscopy after staining with Syto9 and propidium iodide (PI) (1:1) (Figure 4C). PI is a membrane-impermeant dye that only crosses damaged cell membranes and interacts with the cell DNA becoming fluorescent [34]. The dye fluorescence reports on membrane disruption [35]. Thus, we used a LIVE/DEAD BacLight kit assay to determine whether the FSL NPs affect the cell wall integrity of bacteria within the biofilms. The microscopic images showed some individual cells stained in red, confirming that the FSL NPs caused membrane disruption and bacterial death. As it was expected, the empty NPs did not affect the *S. aureus* colonization and establishment of the biofilm.

#### 2.3.2. Elimination of the Mature Biofilm with FSL NPs

**Figure 5:**
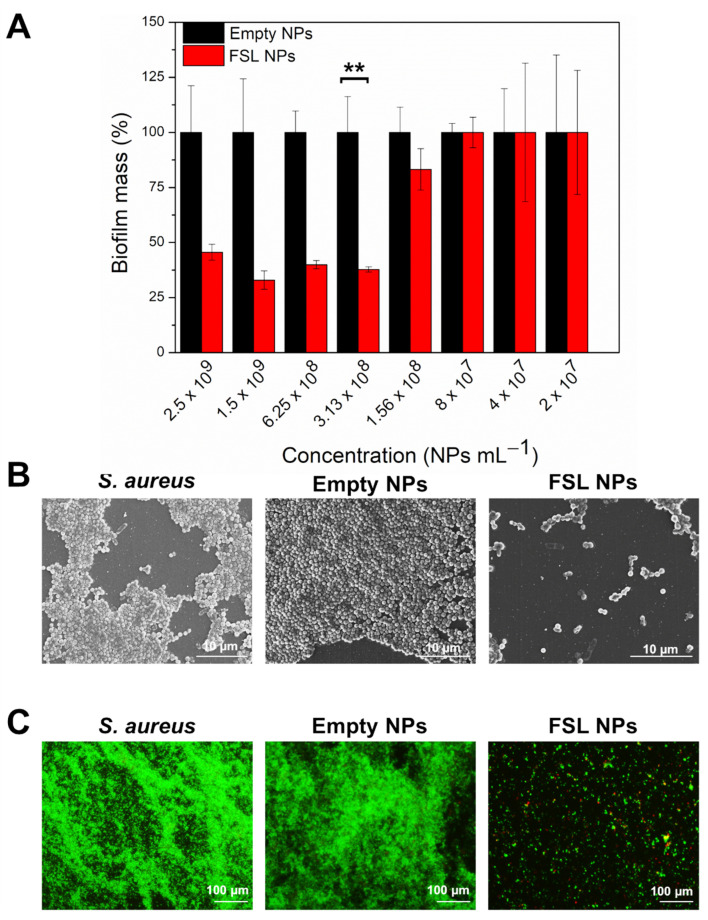
Elimination of *S. aureus* biofilm grown for 24 h. (**A**) Total biofilm quantification using crystal violet assay after treatment with increasing concentration of empty and FSL NPs. (**B**) SEM images of biofilm treated with 3.13 × 10^8^ NPs mL^−1^ empty and FSL NPs. (**C**) LIVE/DEAD BacLight kit microscopic visualization of biofilm treated with 3.13 × 10^8^ NPs mL^−1^ empty and FSL NPs. The green and red fluorescence images are overlaid. The dead bacteria are stained in red and live bacteria are stained in green. Stars represent the statistical differences between the biofilm reduction potential of FSL NPs at the lowest concentration able to reduce the mature biofilm and empty NPs (*p* < 0.001).

Once a mature biofilm is established, the bacteria live in a community, working together to protect themselves against the host immune response and the action of drugs, increasing the MIC of the antimicrobials by 10 to 1000 times, compared to the planktonic cells [36]. Hence, we investigated the biofilm eradication capacity of FSL NPs on an already established *S. aureus* biofilm, quantifying the total biofilm mass composed of the EPM and bacterial cells using a crystal violet assay. Farnesol, in its bulk form, could not eliminate the drug-resistant *S. aureus* biofilm, probably due to its hydrophobicity and poor retention, which restricts its penetration through the biofilm structure (Figure S4). Unlike the empty NPs that did not demonstrate the ability to eliminate the biofilm mass, the nanoformulated farnesol reduced the *S. aureus* biofilm by 60% at a very low concentration of active (Figure 5A). The lowest concentration of FSL NP needed for biofilm reduction was 3.13 × 10^8^ NPs mL^−1^. A lower NP concentration did not affect the biofilm structure, showing that the biofilm elimination properties are concentration-dependent. The FSL NPs could penetrate into the biofilm structure and interact with the sessile *S. aureus* cells causing their death. Farnesol was reported to reduce the amount of proteins and polysaccharides of the EPM, changing the biofilm matrix composition, and promoting biofilm destabilization and biomass reduction [37].

SEM micrographs revealed individually attached cells on the surface and a significant decrease in the biofilm mass after treatment with FSL NPs (Figure 5B), corroborated also by LIVE/DEAD BacLight kit assay (Figure 5C). In contrast, the application of empty NPs neither decreased nor eliminated the *S. aureus* biofilm.

### 2.4. Antiviral Properties of FSL NPs

**Figure 6:**
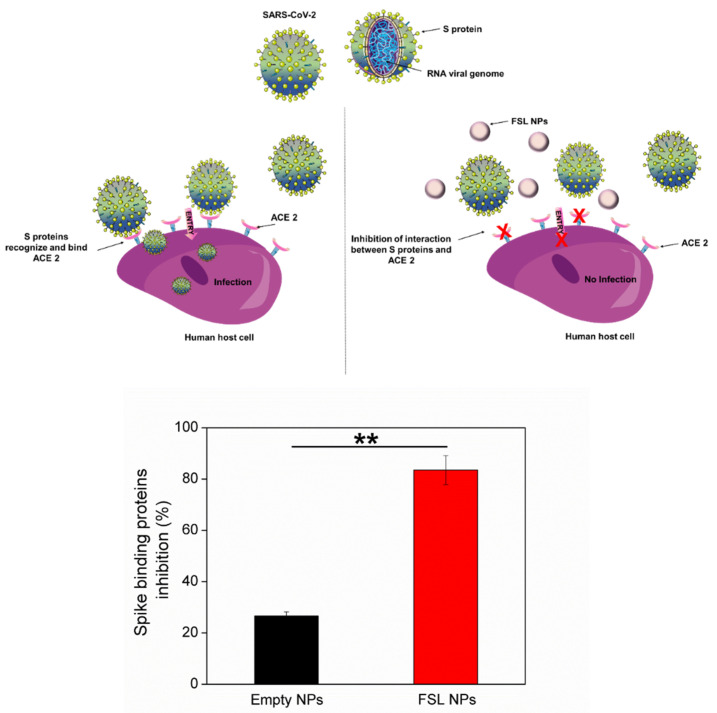
Schematic representation of interaction between the spike-binding proteins of SARS-CoV-2 and the ACE2 receptor on the host cell, and its inhibition after exposure to empty and FSL NPs. Parts of the figure were created using templates from Servier Medical Art (smart.servier.com (accessed on 18 June 2022)). Stars represent the statistical differences between empty and FSL NP inhibition of the spike protein binding interaction and the ACE2 receptor (*p* < 0.001).

The viral infections are initiated by the interaction of the virion and the receptor on the host cell surface. Subsequently, after binding the receptor, the viruses enter into the cells and deliver their genetic information. Various viruses, such as coronaviruses and avian infectious bronchitis virus, fuse and infect the host cells using spike proteins, located on the surface of the viral particle [38,39]. The spike proteins of SARS-CoV-2 recognize ACE2 on the host cell by the receptor-binding domain facilitating the viral attachment and entry into the host cell. Since the spike proteins are critical for the viral entry into the host, targeting the viral–host interaction is an attractive antiviral strategy to manage COVID-19 infections. Based on the lipophilic structure of farnesol and its affinity to interact with microbial membranes, we investigated the ability of the developed FSL NPs to impair the interaction between the spike-protein-binding domain and ACE2. The FSL NPs inhibited the spike protein binding capacity towards ACE2 by 83% (Figure 6). No significant inhibition was observed when empty NPs were applied, confirming that the inhibition properties were due to the presence of farnesol in the NPs. The lipophilic nature of farnesol may intercalate into the double lipid layer of the viral envelope, causing membrane fluidity alterations, and inhibition of virus attachment and further intracellular penetration into the host cell.

### 2.5. In Vitro Assessment of Skin Irritation, Tissue Viability, and Immune Response after Exposure to the FSL NPs

**Figure 7:**
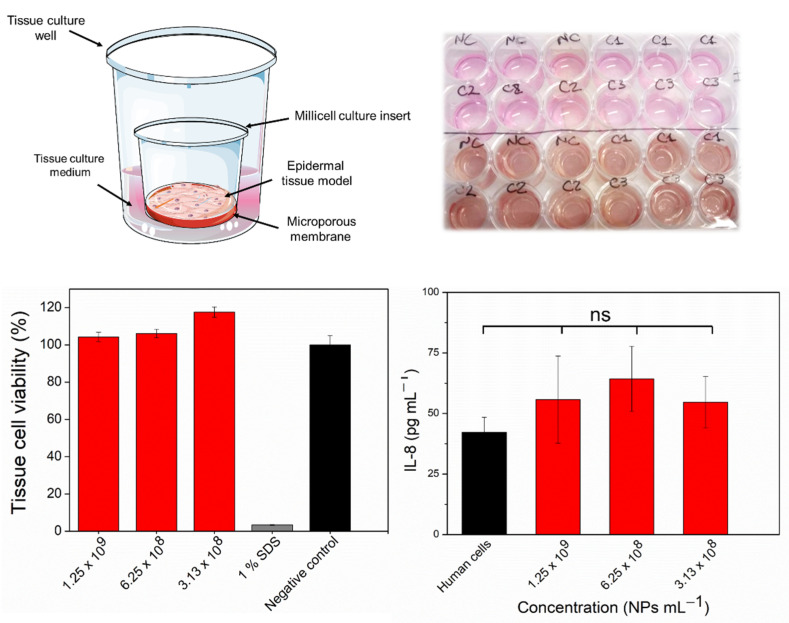
In vitro irritation test using an EpiDerm^TM^ 3D skin model, treated with different concentrations of FSL NP during 18 h exposure. All data are mean values of three independent experiments; ns—statistically not significantly different (*p* > 0.05).

Despite the great antimicrobial properties of FSL NPs, their potential to induce toxicity or an immune response is of important concern for further biomedical application. Thus, the ability of the novel nanoentities to cause skin irritation was studied using a 3D Epiderm™ skin tissue model. In the irritation test, the skin viability after exposure to the positive control (1% (*w/v*) SDS) decreased to 1% (Figure 7). The tissue cell viability was approximately 100% after 18 h exposure to FSL NPs in bactericidal and antibiofilm concentrations compared to the negative control (skin treated with mQ H_2_O), indicating that the NPs are non-irritant.

Since cytokine production is a biomarker for induced immunotoxicity, the potential of the FSL NPs to activate proinflammatory IL-8 cytokine release was assessed. The exposure of skin tissue cells to FSL NPs at bactericidal concentrations did not significantly promote undesirable immunostimulation associated with high levels of proinflammatory IL-8, compared to the cells without treatment.

## 3. Materials and Methods

### 3.1. Materials

Farnesol solution (95% (*v/v*)) was purchased from Sigma-Aldrich (Madrid, Spain). The monobutyl ester of poly (methyl vinyl ether/maleic) acid was kindly provided from Bionanoplus (Navarra, Spain). The bacterial strain *S. aureus* (ATCC 25923) was obtained from the American Type Culture Collection (ATCC LGC Standards, Barcelona, Spain). SARS-CoV-2 Spike: ACE2 Inhibitor Screening Assay Kit—96 reactions were provided from Quimigen S.L. (Madrid, Spain). A reconstructed EpiDerm™ human 3D skin tissue model was purchased from MatTek Corporation (Ashland, MA, USA) and an IL-8 ELISA matched antibody pair kit was obtained from Invitrogen, Life Technologies (Monza, Italy). Ultrapure water (mQ H_2_O with 18.2 MΩ cm resistivity) was used in all experiments. All other chemical and microbiological reagents were purchased from Sigma-Aldrich, unless otherwise specified.

### 3.2. FSL NPs Preparation

Under magnetic stirring, 6 g of poly (methyl vinyl ether/maleic) acid polymer was mixed with 44 g of propylene glycol. Then, a two-phase solution comprised of a 15 mL polymer solution, containing 3% (*v/v*) farnesol (organic phase) and 15 mL mQ H_2_O was placed into a thermostated sonicator cell (20 ± 1 °C). The same biphasic system without farnesol was used to obtain the empty control NPs. The NPs were synthesized with a high- intensity Vibra-Cell VCX 750 ultrasonic processor (Sonics and Materials, Inc., Newtown, CT, USA) using 20 kHz Ti horn at 50% amplitude for 12 min. The resultant NP dispersion was purified by centrifugation using 100 kDa Amicon centrifuge filters at 4000× *g* for 20 min to remove the non-encapsulated farnesol.

### 3.3. NPs Characterization

The zeta-potential and the PDI of the empty and farnesol NPs (FSL NPs) was determined using a Zetasizer Nano ZS (Malvern Instruments Inc., Malvern, UK). The NP concentration (NPs per mL), mean size, and NP size distribution were evaluated by nanoparticle tracking analysis (NTA) using a NanoSight NS 300 (Malvern Instruments Inc., Malvern, UK) in flow mode, and the software NTA 3.2 to capture several frames of the NP suspension, obtaining the final concentration and the hydrodynamic diameter of the NPs. The size and the morphology of the NPs were further examined by scanning transmission electron microscope (STEM) (ICN2 XHRSEM Magellan 400 L) at a 20 kV acceleration voltage.

The concentration of farnesol loaded into the NPs was determined using high performance liquid chromatography (HPLC) (Series 1200, Agilent Technologies, Barcelona, Spain)) with a Kromasil^®^ Eternity™ C18 column (50 × 4.6 mm, 5 μm particle size (Supelco, Bellefonte, PA, USA)), at flow rate of 0.5 mL min^−1^, with a gradient of 10% to 90% of MeOH/H_2_O over 20 min, and detection by UV absorbance (210 nm), following a procedure described before [40].

### 3.4. Antibacterial Activity of FSL NPs

#### 3.4.1. Minimum Inhibitory Concentration

The antibacterial activity of FSL NPs was evaluated against the Gram-positive *S. aureus*, as previously described [41]. Briefly, 50 µL of the NPs at different concentrations were placed into a 96-well polypropylene microplate; then, 50 µL of bacterial inoculum in Mueller Hinton broth (MHB) medium, (OD_600_ = 0.01, concentration of ≈5 × 10^6^ CFU mL^−1^) was added. Thereafter, the samples were incubated for 24 h at 37 °C with shaking at 230 rpm; then, bacterial growth in the presence of NPs was assessed measuring the OD at 600 nm in a microplate reader (Infinite M200, Tecan, Grödig, Austria). To determine the survived bacteria and MBC, 15 μL of a bacterial suspension treated with NPs (concentrations corresponding to the MICs) were plated on selective Baird Parker agar and incubated at 37 °C for 24 h.

#### 3.4.2. Bacteria Time-Killing Kinetics

The minimal time required for the NPs to eradicate the pathogen was assessed using a time-killing kinetics assay. The bacteria were grown in MHB at 37 °C at 230 rpm overnight and the bacterial suspensions were diluted in sterile phosphate-buffered saline (PBS) to an OD_600_ = 0.01. Subsequently, 200 μL of the bacteria was incubated with 200 μL of the NPs at bactericidal concentrations, and the samples were incubated at 37 °C under agitation at 230 rpm. The survived bacteria were enumerated after 1, 2, and 3 h using the drop plate method [20].

#### 3.4.3. Scanning Electron Microscopy of Bacteria

The interaction of FSL NPs with the *S. aureus* cultures was evaluated with scanning electron microscopy (SEM). The NPs at MIC were mixed with a *S. aureus* inoculum in PBS (OD = 0.01 at 600 nm), and the samples were incubated for 3 h at 37 °C with shaking. Immediately after the treatment, the samples were centrifuged, washed twice with sterile PBS, and resuspended in 50 μL PBS. Afterward, 15 μL of the suspension was spread on a glass slide and air-dried. The cells were fixed in 4% (*v/v*) formaldehyde overnight, washed with sterile PBS, and sequentially treated with 25, 50, 75, and 96% (*v/v*) EtOH for 10 min each. The samples were coated with sputter gold with a layer thickness of approximately 5–10 nm and observed by SEM Zeiss Axioplan.

#### 3.4.4. Resistance Development Assay

The ability of the FSL NPs to cause resistance development among pathogenic bacteria was evaluated by first determining the MIC value using *S. aureus* ATCC, as described previously, with some modifications [42]. The stock solution of the NPs was diluted in two-fold serial dilutions in LB medium in a 96-well plate (Greiner Bioone). Each well contained 10^5^ CFU mL^−1^ of bacteria, and bacteria treated with double-distilled H_2_O served as a negative control. The bacterial growth was monitored via absorbance measurements at OD_595_ taken with a microplate reader (Synergy 2, BioTek instruments). On the following day, each of the bacteria were serially passaged in two-fold antibiotic ciprofloxacin or NP gradients in a 96- well plate, performing a MIC assay, as described above, except that the bacteria concentration was set at 10^5^ CFU mL^−1^ from the second growth cycle. At the end of each growth cycle (20–24 h), following determination of the MIC, the culture of the highest drug concentration having turbidity, suggestive of bacterial growth, was taken and diluted 1:50. The newly diluted bacterial suspension was grown overnight in a new 96-well plate, conducting a new MIC assay, following which, the absorbance was monitored. This assay was conducted daily for a period of 30 days to determine a change in the MIC value of the antibiotic or the NPs.

### 3.5. Inhibition of S. aureus Attachment and Establishment of Biofilm

#### 3.5.1. Biofilm Inhibition Activity

FSL NPs were tested to determine their minimal inhibitory biofilm concentration against Gram-positive *S. aureus.* The bacteria were grown in tryptic soy broth (TSB) overnight at 37 °C at 230 rpm. Then, 50 µL of the antibacterial compounds were mixed with 50 µL of the bacterial suspension (OD _600_ = 0.01) and left to grow for 24 h at 37 °C without shaking (static culture). Subsequently, the total biofilm mass was quantified using a crystal violet assay, as previously described [41]. The wells were washed three times with 200 µL distilled H_2_O and the biofilms were left for 60 min at 60 °C for biofilm fixation. Further, the biofilm was stained for 5–10 min with 200 µL of a 0.1% (*w/v*) crystal violet solution, washed with distilled H_2_O, and dried at 60 °C. Then, 200 µL of 30% (*v/v*) acetic acid was added to each well to dissolve the crystal violet. A total of 125 µL from each well was transferred to a 96-well plate and the absorbance was measured at 595 nm.

The biofilm inhibition of FSL NPs was observed using SEM. After incubation with NPs, the biofilms were washed using sterile PBS and the formed biofilms were fixed with 4% (*v/v*) formaldehyde overnight. Subsequently, the biofilms were washed with sterile PBS and treated with 25, 50, 75, and 96% (*v/v*) ethanol for 10 min each. The samples were left to dry at RT and observed using a SEM Zeiss Axioplan microscope.

The live and dead bacteria into the biofilms were further observed with fluorescence microscopy using a LIVE/DEAD BacLight kit [41]. After the incubation, the planktonic cells were washed three times with 200 µL sterile PBS (pH = 7.4), and the microplate was incubated with 20 µL of SYTO9 and PI in a ratio of 1:1. Further, the samples were visualized under fluorescence microscopy using a 20× objective lens. The live cells were stained in green and the dead ones in red.

#### 3.5.2. FSL NP Interaction with Mature Biofilms

The ability of the NPs to eradicate the established biofilm of *S. aureus* was assessed using a crystal violet assay, as previously described [41]. Briefly, 100 µL of overgrown bacteria, diluted in TSB (OD_600_ = 0.01) was left for 24 h at 37 °C at static conditions to form; then, the non-adhered bacterial cells were washed with sterile 100 mM PBS (pH 7.4). Subsequently, 60 µL of the antibacterial compound at different concentrations were mixed with 60 µL of the medium and the microplate was incubated for 24 h at 37 °C. The biofilms were washed three times with 200 µL distilled H_2_O and further incubated for 60 min at 60 °C for biofilm fixation. The biofilm was stained for 5–10 min with 200 µL of a 0.1% (*w/v*) crystal violet solution and then washed three times with distilled H_2_O. The plate was dried at 60 °C and 200 µL of 30% (*v/v*) acetic acid was added to each well to dissolve the crystal violet. A total of 125 µL from each well was transferred to a 96-well plate and the absorbance was measured at 595 nm. The mature biofilms were further observed using SEM and a LIVE/DEAD kit assay as described above.

### 3.6. SARS-CoV-2 Spike: ACE2 Inhibitor Screening Assay Kit

The anti-viral properties of FSL NPs were validated as a percentage of SARS-CoV-2 spike protein binding to the ACE2 in the presence of the FSL NPs using an ACE2:SARS-CoV-2 spike inhibitor screening assay kit (Quimigen S.L. #79931; Madrid, Spain) according to the protocol described in the manufacturer’s datasheet. The samples were read in a microtiter plate (Infinite M200, Tecan, Grödig, Austria) capable of reading chemiluminescence.

### 3.7. Biocompatibility Assessment

#### 3.7.1. In Vitro Irritation Assay and Tissue Viability after Exposure to the FSL NPs

The biocompatibility of the developed bactericidal nanoentities was evaluated using EpiDerm^TM^ 3D tissue models, as previously described [43]. Briefly, the EpiDerm^TM^ 3D tissue models were exposed to 100 µL of FSL NPs at different concentrations (3.13 × 10^8^ NPs mL^−1^, 1.56 × 10^8^ NPs mL^−1^, 8 × 10^7^ NPs mL^−1^) for 18 h. Sodium dodecyl sulfate (1% (*w/v*) SDS solution) was used for the positive control. After exposure, the test materials were removed by repeated rinsing with PBS and the skin models were transferred to the new plates for viability testing. Then, the skin model was transferred into a 24-well plate for the cell viability test and 300 μL of 3-(4,5-dimethylthiazol-2-yl)-2.5-diphenyltetrazolium bromide (MTT) solution (0.3 mg mL^−1^) was added to each skin model. The skins were incubated in a 5% CO_2_ incubator for 3 h and insoluble formazan products of MTT were extracted with 2 mL isopropanol per well. Subsequently, 200 μL of the extract was transferred onto a 96-well plate to measure the OD at 570 nm.

#### 3.7.2. Immunotoxicity Induction by FSL NPs

As a marker of the pro-inflammatory potential, the release of interleukin-8 (IL-8) by the skin model was assessed. Subsequently, after 18 h of NP exposure to the skin model, the medium was collected and the samples were frozen at −20 °C until use. Human IL-8 CytoSet ELISA kits (Invitrogen, Life Technologies, Monza, Italy) were used according to the manufacturer’s protocol. Briefly, 96-well plates were coated with capture antibody and incubated overnight at room temperature. Supernatants were added to the plate and incubated for 2 h. Then, the detection antibody was loaded into each well for a further incubation. Finally, horseradish-conjugated streptavidin and the peroxidase substrate were added. The reaction was stopped by 1.8 N H_2_SO_4_, and OD was measured with a microplate spectrophotometer (Infinite 200 Pro, TECAN, Männedorf, Switzerland) at 450 nm, using 650 nm as a reference wavelength. The results represent the mean of three independent experiments ± SE.

### 3.8. Statistical Analysis

All data are presented as mean ± standard deviation. For multiple comparisons, statistical analysis by a one-way analysis of variance (ANOVA) followed by post-hoc Tukey’s test or the unpaired two-tailed Student’s *t*-test method, were carried out using Graph Pad Prism Software 5.04 for windows (USA). The *p* values less than 0.05 were considered as statistically significant.

## 4. Conclusions

The spread of microbial infections and AMR demand the development of novel effective strategies for controlling the human pathogens and prevent the occurrence of drug resistance. In this work, farnesol was converted into efficient antimicrobial NPs using one-step high intensity US. The nanoformulation imparted farnesol with enhanced antibacterial properties at a very low concentration, compared to its bulk form. The FSL NPs eliminated *S. aureus* within less than 3 h of contact due to improved interaction with the bacterial membrane, minimizing the appearance of drug resistance. Farnesol, in its nano-form, was able to both impede the establishment of biofilms and eradicate mature biofilms. The FSL NPs inhibited the interaction of SARS-CoV-2 binding proteins with ACE2 receptors on host cells by 83% in vitro, demonstrating promising antiviral activity. Moreover, the FSL NPs did not affect the cell viability in a 3D tissue cell model and did not induce immunotoxicity, confirming their biocompatibility. Together, these results reveal the therapeutic potential of the novel nanomodalities for the prevention and treatment of microbial infections without inducing the occurrence of resistance. Their application may be extended to other biomedical applications such as coatings on high-touch surfaces, personal protective equipment, and medical devices.

## Data Availability

All data generated or analyzed during this study are included in this manuscript and its Supplementary Materials.

## Supplementary Materials

The following supporting information can be downloaded at: https://www.mdpi.com/article/10.3390/ijms23147527/s1.
