# Supplementary material for: Antibacterial, Antibiofilm, and Antiviral Farnesol-Containing Nanoparticles Prevent Staphylococcus aureus from Drug Resistance Development
Source: Int J Mol Sci. 2022 Jul 7;23(14):7527. doi: 10.3390/ijms23147527 (PMC9321328; doi:10.3390/ijms23147527)
Supplement: Supplementary file 1 [file ijms-23-07527-s001.zip › ijms-1802924-supplementary.pdf]

Supplementary Materials

Antibacterial, Antibiofilm, and Antiviral Farnesol-Containing Nanoparticles Prevent *Staphylococcus aureus* from Drug Resistance Development

Aleksandra Ivanova [1], Kristina Ivanova [1], Luisa Fiandra [2], Paride Mantecca [2], Tiziano Catelani [3], Michal Natan [4], Ehud Banin [4], Gila Jacobi [4], and Tzanko Tzanov [1,*]

[1] Group of Molecular and Industrial Biotechnology, Chemical engineering, Universitat Politécnica de Catalunya, Terrassa, Spain; aleksandra.asenova@upc.edu (A.I.); kristina.ivanova@upc.edu (K.I.);

[2] Department of Earth and Environmental Sciences, Research Center POLARIA, Universita degli Studi di Milano-Bicocca, Milano, Italy; luisa.fiandra@unimib.it (L.F.); paride.mantecca@unimib.it (P.M.);

[3] Interdepartmental Microscopy Platform, University of Milano – Bicocca, Milano, Italy; tiziano.catelani@unimib.it (T.C.);

[4] The Institute for Advanced Materials and Nanotechnology, The Mina and Everard Goodman Faculty of Life Sciences, Bar-Ilan University, Ramat-Gan 52900, Israel; natan.michal@gmail.com (M.N.); Ehud.Banin@biu.ac.il (E.B.); gilajacobi@gmail.com (G.J.);

* Correspondence: tzanko.tzanov@upc.edu ;Tel.: (optional; include country code; if there are multiple corresponding authors, add author initials)

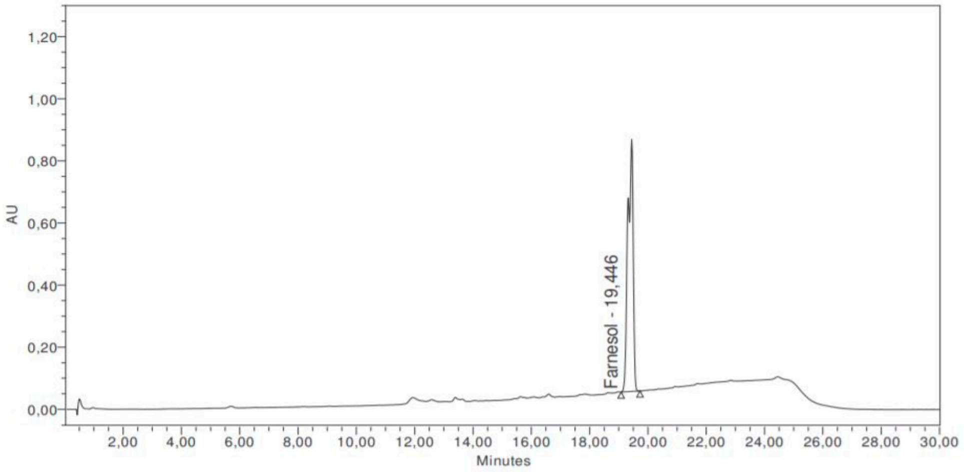

Figure S1. HPLC chromatogram of farnesol into FSL NPs.

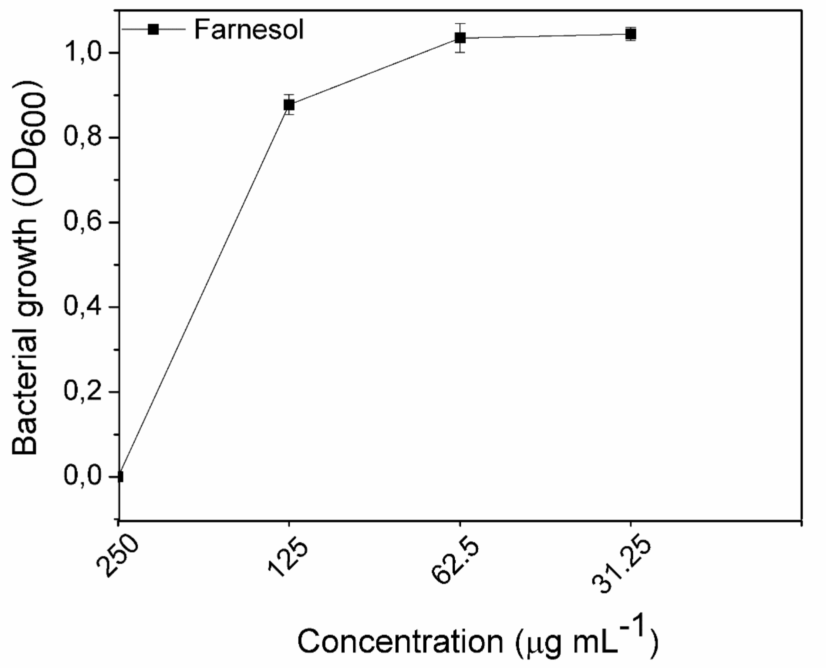

Figure S2. Antibacterial activity of farnesol bulk solution.

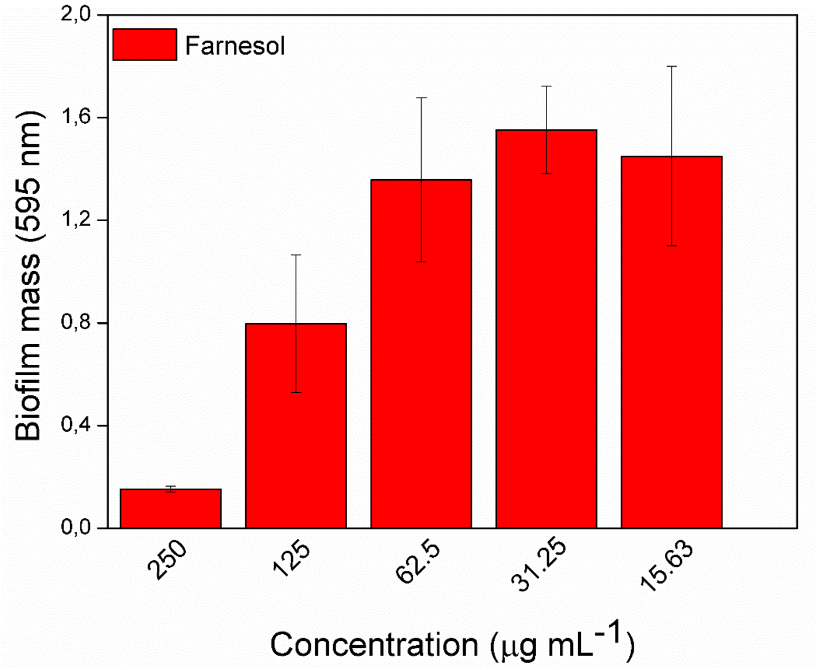

Figure S3. Biofilm inhibition properties of farnesol bulk solution.

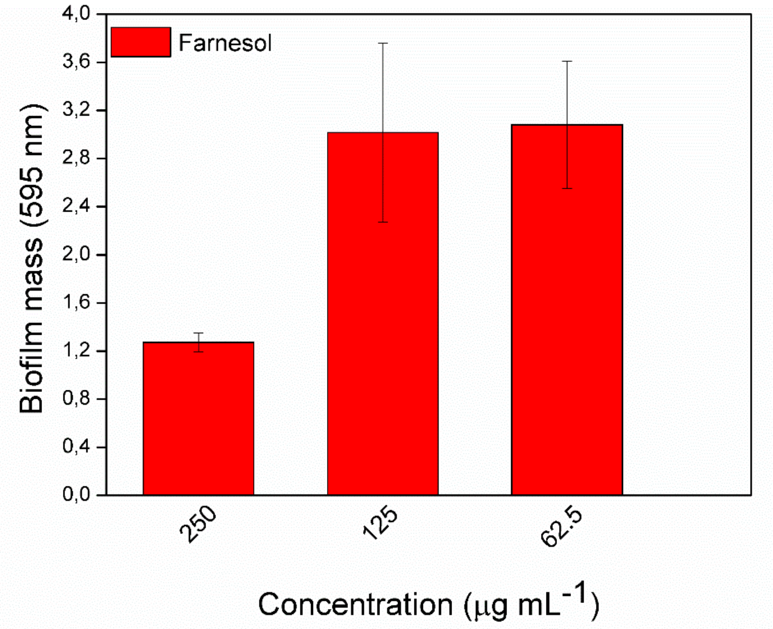

Figure S4. Biofilm eradication properties of farnesol bulk solution.
